# 1-Benzoyl-3-(naphthalen-1-yl)thio­urea

**DOI:** 10.1107/S160053681104582X

**Published:** 2011-11-05

**Authors:** Sohail Saeed, Naghmana Rashid, Jerry P Jasinski, James A Golen

**Affiliations:** a Department of Chemistry, Research Complex, Allama Iqbal Open University, Islamabad 44000, Pakistan; b Department of Chemistry, Keene State College, 229 Main Street, Keene, NH 03435-2001, USA

## Abstract

In the title compound, C_18_H_14_N_2_OS, the dihedral angle between the mean planes of the 3-naphthyl and 1-benzoyl rings is 20.7 (1)°. The crystal packing is stabilized by weak N—H⋯S inter­actions. Intra­molecular N—H⋯O and C—H⋯O hydrogen bonding is also observed.

## Related literature

For the biological activity of thio­urea in medicinal chemistry, see: Saeed *et al.* (2009 ▶, 2010*a* ▶,*b* ▶); Maddani & Prabhu (2010 ▶). For the use of thio­urea derivatives in organocatalysis, see: Jung & Kim (2008 ▶) and for their use as curing agents for ep­oxy resins, see: Saeed *et al.* (2011 ▶). For the use of thio­ureas as ligands in coordination chemistry, see: Burrows *et al.* (1999 ▶); Henderson *et al.* (2002 ▶); Schuster *et al.* (1990 ▶). For the pesticidal activity of acyl thio­ureas, see: Che *et al.* (1999 ▶). For standard bond lengths, see Allen *et al.* (1987 ▶).
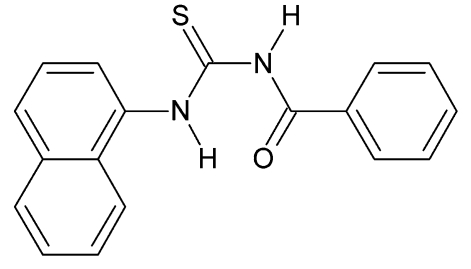


## Experimental

### 

#### Crystal data

C_18_H_14_N_2_OS
*M* *_r_* = 306.37
Monoclinic, 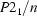

*a* = 9.7368 (14) Å
*b* = 5.2256 (10) Å
*c* = 28.619 (4) Å
β = 92.126 (12)°
*V* = 1455.2 (4) Å^3^
*Z* = 4
Mo *K*α radiation
μ = 0.23 mm^−1^
*T* = 173 K
0.35 × 0.08 × 0.08 mm

#### Data collection

Oxford Diffraction Xcalibur Eos Gemini diffractometer
Absorption correction: multi-scan (*CrysAlis RED*; Oxford Diffraction, 2010 ▶) *T*_min_ = 0.925, *T*_max_ = 0.982
12731 measured reflections
3460 independent reflections
2206 reflections with *I* > 2σ(*I*)
*R*_int_ = 0.082

#### Refinement

*R*[*F* ^2^ > 2σ(*F* ^2^)] = 0.062
*wR*(*F* ^2^) = 0.135
*S* = 1.05
3460 reflections
205 parameters
2 restraints
H atoms treated by a mixture of independent and constrained refinement
Δρ_max_ = 0.25 e Å^−3^
Δρ_min_ = −0.36 e Å^−3^

### 

Data collection: *CrysAlis PRO* (Oxford Diffraction, 2010 ▶); cell refinement: *CrysAlis PRO*; data reduction: *CrysAlis RED* (Oxford Diffraction, 2010 ▶); program(s) used to solve structure: *SHELXS97* (Sheldrick, 2008 ▶); program(s) used to refine structure: *SHELXL97* (Sheldrick, 2008 ▶); molecular graphics: *SHELXTL* (Sheldrick, 2008 ▶); software used to prepare material for publication: *SHELXTL*.

## Supplementary Material

Crystal structure: contains datablock(s) global, I. DOI: 10.1107/S160053681104582X/fk2043sup1.cif

Structure factors: contains datablock(s) I. DOI: 10.1107/S160053681104582X/fk2043Isup2.hkl

Supplementary material file. DOI: 10.1107/S160053681104582X/fk2043Isup3.cml

Additional supplementary materials:  crystallographic information; 3D view; checkCIF report

## Figures and Tables

**Table 1:** Hydrogen-bond geometry (Å, °)

*D*—H⋯*A*	*D*—H	H⋯*A*	*D*⋯*A*	*D*—H⋯*A*
N2—H2⋯O1	0.86 (2)	1.85 (2)	2.600 (3)	144 (2)
N1—H1⋯S1^i^	0.86 (2)	2.80 (2)	3.591 (2)	153 (2)
C15—H15*A*⋯O1	0.95	2.51	3.411 (3)	159

Symmetry code: (i) 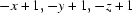.

**Fig. 1.**
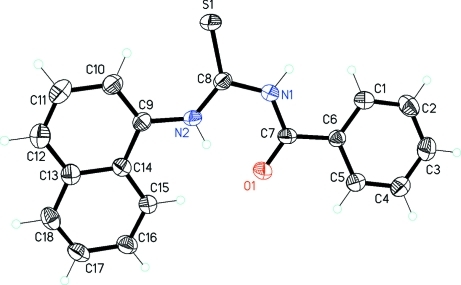
Molecular structure of the title compound showing the atom labeling scheme and 50% probability displacement ellipsoids.

**Fig. 2.**
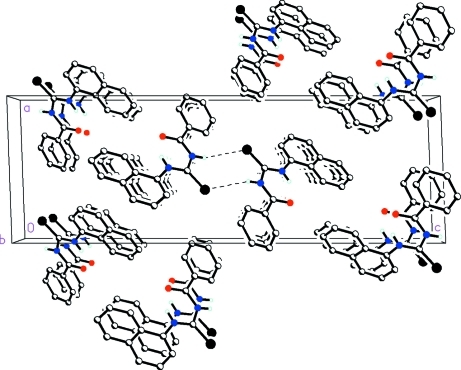
Packing diagram of the title compound viewed along the b axis. Dashed lines indicate weak N1—H1···S1 intermolecular interactions.

## supplementary crystallographic information

### Comment

Thioureas are the subject of significant interest because of their usefulness in medicinal chemistry due to their biological activity as fungicides (Saeed *et al.*, 2010*a*), anticancer (Saeed *et al.*, 2010*b*),herbicides, rodenticides and phenoloxidase enzymatic inhibitors (Maddani & Prabhu, 2010). Recently, thiourea derivatives have found use in organocatalysis (Jung & Kim, 2008). Amino-thiourea derivatives (Saeed *et al.*, 2009) and their transition metal complexes are used as curing agents for epoxy resins (Saeed *et al.*, 2011). Thioureas have a long history as a ligand in coordination chemistry and coordinate readily to a metal *via* sulfur and oxygen (Burrows *et al.*, 1999). These hard and soft donor atoms provide a multitude of bonding possibilities (Henderson *et al.*, 2002). Hydrogen bonding behavior of some thioureas have been investigated and it is found that intramolecular hydrogen bonds between the carbonyl oxygen and a nitrogen atom is common. The complexing capacity of thiourea derivatives has been reported (Schuster *et al.*, 1990). Also, some acyl thioureas have been found to possess pesticidal activities and promote plant growth while others have been shown to have a notable positive effect on the germination of maize seeds and on the chlorophyll contents in seedling leaves (Che *et al.*, 1999). With the simultaneous presence of S, N and O electron donors, the versalitility and behavior of acylthioureas as building blocks in polydentate ligands for metal ions have become a recent topic of interest. Substituted acylthiourea ligands might act as monodentate sulfur donors, bidentate oxygen and nitrogen donors. In continuation of our research program concerned with structural modification of biologically active thiourea derivatives and their transition metal complexes, we aim to incorporate the aliphatic and aromatic moieties in the substituted phenyl nucleus with thiourea functionality to obtain new functions in an attempt to improve the antimicrobial profile of these compounds. In view of the importance of thiourea derivatives, the crystal structure of the title compound, C_18_H_14_N_2_O_S_, (I), is reported.

In the title compound, (I), the dihedral angle between the mean planes of the 3-naphthyl and 1-benzoyl rings is 20.7 (1)° (Fig. 1). Crystal packing is stabilized by weak N1—H1···S1 intermolecular interactions (Table 1, Fig. 2). N2—H2···O1 intramolecular hydrogen bonds are also observed (Table 1).

### Experimental

A solution of benzoyl chloride (0.01 mol) in anhydrous acetone (80 ml) and 3% tetrabutylammonium bromide (TBAB) as a phase-transfer catalyst (PTC) in anhydrous acetone was added dropwise to a suspension of dry ammonium thiocyanate (0.01 mol) in acetone (50 ml) and the reaction mixture was refluxed for 45 min. After cooling to room temperature, a solution of 1-naphthylamine (0.01 mol) in anhydrous acetone (25 ml) was added dropwise and the resulting mixture refluxed for 2.5 h. Hydrochloric acid (0.1 N, 300 ml) was added, and the solution was filtered. The solid product was washed with water and purified by re-crystallization from ethanol.

### Refinement

All H atoms were positioned with idealized geometry using a riding model, [C—H = 0.95Å and *U*_iso_ = 1.2*U*_eq_(C,N)]. H(N) positions were refined freely.

### Crystal data

**Table:**

C_18_H_14_N_2_OS	*F*(000) = 640
*M**_r_* = 306.37	*D*_x_ = 1.398 Mg m^−^^3^
Monoclinic, *P*2_1_/*n*	Mo *K*α radiation, λ = 0.71073 Å
Hall symbol: -P 2yn	Cell parameters from 1307 reflections
*a* = 9.7368 (14) Å	θ = 3.5–32.3°
*b* = 5.2256 (10) Å	µ = 0.23 mm^−^^1^
*c* = 28.619 (4) Å	*T* = 173 K
β = 92.126 (12)°	Rod, colourless
*V* = 1455.2 (4) Å^3^	0.35 × 0.08 × 0.08 mm
*Z* = 4	

### Data collection

**Table:**

Oxford Diffraction Xcalibur Eos Gemini diffractometer	3460 independent reflections
Radiation source: Enhance (Mo) X-ray Source	2206 reflections with *I* > 2σ(*I*)
graphite	*R*_int_ = 0.082
Detector resolution: 16.1500 pixels mm^-1^	θ_max_ = 27.9°, θ_min_ = 4.0°
ω scans	*h* = −12→12
Absorption correction: multi-scan (*CrysAlis RED*; Oxford Diffraction, 2010)	*k* = −6→6
*T*_min_ = 0.925, *T*_max_ = 0.982	*l* = −34→37
12731 measured reflections	

### Refinement

**Table:**

Refinement on *F*^2^	Primary atom site location: structure-invariant direct methods
Least-squares matrix: full	Secondary atom site location: difference Fourier map
*R*[*F*^2^ > 2σ(*F*^2^)] = 0.062	Hydrogen site location: inferred from neighbouring sites
*wR*(*F*^2^) = 0.135	H atoms treated by a mixture of independent and constrained refinement
*S* = 1.05	*w* = 1/[σ^2^(*F*_o_^2^) + (0.0427*P*)^2^ + 0.1745*P*] where *P* = (*F*_o_^2^ + 2*F*_c_^2^)/3
3460 reflections	(Δ/σ)_max_ = 0.001
205 parameters	Δρ_max_ = 0.25 e Å^−^^3^
2 restraints	Δρ_min_ = −0.36 e Å^−^^3^

### Special details

**Table:**

Geometry. All e.s.d.'s (except the e.s.d. in the dihedral angle between two l.s. planes) are estimated using the full covariance matrix. The cell e.s.d.'s are taken into account individually in the estimation of e.s.d.'s in distances, angles and torsion angles; correlations between e.s.d.'s in cell parameters are only used when they are defined by crystal symmetry. An approximate (isotropic) treatment of cell e.s.d.'s is used for estimating e.s.d.'s involving l.s. planes.
Refinement. Refinement of *F*^2^ against ALL reflections. The weighted *R*-factor *wR* and goodness of fit *S* are based on *F*^2^, conventional *R*-factors *R* are based on *F*, with *F* set to zero for negative *F*^2^. The threshold expression of *F*^2^ > σ(*F*^2^) is used only for calculating *R*-factors(gt) *etc*. and is not relevant to the choice of reflections for refinement. *R*-factors based on *F*^2^ are statistically about twice as large as those based on *F*, and *R*- factors based on ALL data will be even larger..

### Fractional atomic coordinates and isotropic or equivalent isotropic displacement parameters (Å^2^)

**Table:**

	*x*	*y*	*z*	*U*_iso_*/*U*_eq_	
S1	0.63172 (7)	0.32704 (17)	0.54848 (3)	0.0446 (2)	
O1	0.28206 (18)	0.5576 (4)	0.63936 (6)	0.0395 (5)	
N1	0.4117 (2)	0.5657 (4)	0.57512 (7)	0.0300 (5)	
H1	0.423 (3)	0.632 (5)	0.5480 (7)	0.036*	
N2	0.4889 (2)	0.2542 (4)	0.62654 (7)	0.0280 (5)	
H2	0.421 (2)	0.315 (5)	0.6413 (9)	0.034*	
C1	0.2515 (3)	1.0084 (5)	0.54374 (9)	0.0313 (6)	
H1A	0.3367	0.9825	0.5293	0.038*	
C2	0.1632 (3)	1.1966 (5)	0.52738 (10)	0.0413 (7)	
H2A	0.1872	1.2993	0.5016	0.050*	
C3	0.0399 (3)	1.2359 (6)	0.54847 (11)	0.0427 (7)	
H3A	−0.0216	1.3642	0.5369	0.051*	
C4	0.0059 (3)	1.0899 (6)	0.58617 (10)	0.0407 (7)	
H4A	−0.0784	1.1195	0.6010	0.049*	
C5	0.0929 (3)	0.9015 (5)	0.60260 (10)	0.0364 (7)	
H5A	0.0685	0.8010	0.6287	0.044*	
C6	0.2167 (2)	0.8566 (5)	0.58122 (8)	0.0257 (5)	
C7	0.3049 (2)	0.6496 (5)	0.60124 (8)	0.0272 (6)	
C8	0.5082 (2)	0.3745 (5)	0.58638 (9)	0.0292 (6)	
C9	0.5611 (2)	0.0543 (5)	0.64982 (9)	0.0268 (6)	
C10	0.6612 (2)	−0.0889 (5)	0.62991 (9)	0.0331 (6)	
H10A	0.6876	−0.0526	0.5990	0.040*	
C11	0.7248 (3)	−0.2889 (6)	0.65515 (10)	0.0395 (7)	
H11A	0.7964	−0.3833	0.6415	0.047*	
C12	0.6859 (3)	−0.3498 (5)	0.69861 (10)	0.0379 (7)	
H12A	0.7289	−0.4890	0.7147	0.046*	
C13	0.5827 (2)	−0.2094 (5)	0.72029 (9)	0.0297 (6)	
C14	0.5195 (2)	−0.0002 (5)	0.69628 (8)	0.0261 (5)	
C15	0.4192 (2)	0.1399 (5)	0.71991 (9)	0.0306 (6)	
H15A	0.3758	0.2818	0.7048	0.037*	
C16	0.3832 (3)	0.0759 (5)	0.76395 (9)	0.0354 (7)	
H16A	0.3159	0.1740	0.7791	0.043*	
C17	0.4444 (3)	−0.1323 (5)	0.78703 (9)	0.0371 (7)	
H17A	0.4181	−0.1770	0.8176	0.045*	
C18	0.5413 (3)	−0.2703 (5)	0.76558 (9)	0.0339 (6)	
H18A	0.5826	−0.4119	0.7815	0.041*	

### Atomic displacement parameters (Å^2^)

**Table:**

	*U*^11^	*U*^22^	*U*^33^	*U*^12^	*U*^13^	*U*^23^
S1	0.0376 (4)	0.0694 (6)	0.0275 (4)	0.0166 (4)	0.0126 (3)	0.0087 (4)
O1	0.0407 (11)	0.0504 (12)	0.0283 (11)	0.0153 (10)	0.0124 (8)	0.0099 (9)
N1	0.0283 (11)	0.0381 (13)	0.0239 (12)	0.0030 (10)	0.0044 (9)	0.0055 (10)
N2	0.0248 (11)	0.0373 (13)	0.0223 (11)	0.0044 (10)	0.0066 (9)	0.0002 (9)
C1	0.0320 (14)	0.0316 (15)	0.0304 (15)	−0.0021 (12)	0.0043 (11)	−0.0020 (12)
C2	0.0535 (18)	0.0349 (16)	0.0356 (16)	0.0020 (14)	0.0032 (14)	0.0069 (13)
C3	0.0403 (16)	0.0390 (17)	0.0485 (19)	0.0075 (14)	−0.0042 (14)	0.0044 (14)
C4	0.0312 (15)	0.0450 (18)	0.0460 (18)	0.0060 (14)	0.0049 (13)	0.0053 (14)
C5	0.0315 (14)	0.0396 (17)	0.0384 (16)	0.0038 (13)	0.0063 (12)	0.0069 (13)
C6	0.0249 (13)	0.0277 (14)	0.0245 (13)	−0.0005 (11)	0.0015 (10)	−0.0014 (11)
C7	0.0261 (13)	0.0317 (14)	0.0241 (13)	−0.0010 (11)	0.0055 (10)	−0.0017 (11)
C8	0.0233 (13)	0.0381 (16)	0.0260 (14)	0.0033 (12)	0.0009 (10)	−0.0009 (12)
C9	0.0237 (12)	0.0276 (14)	0.0289 (14)	−0.0001 (11)	0.0003 (10)	−0.0024 (11)
C10	0.0285 (14)	0.0390 (16)	0.0319 (15)	0.0033 (12)	0.0017 (11)	−0.0021 (12)
C11	0.0312 (15)	0.0408 (17)	0.0463 (18)	0.0070 (13)	−0.0002 (13)	−0.0087 (14)
C12	0.0376 (15)	0.0348 (16)	0.0409 (17)	0.0019 (13)	−0.0050 (13)	0.0004 (13)
C13	0.0276 (13)	0.0270 (14)	0.0340 (15)	−0.0040 (11)	−0.0040 (11)	−0.0003 (11)
C14	0.0258 (13)	0.0260 (13)	0.0263 (14)	−0.0058 (11)	−0.0010 (10)	−0.0011 (10)
C15	0.0305 (14)	0.0312 (14)	0.0301 (14)	0.0025 (12)	0.0022 (11)	0.0024 (11)
C16	0.0394 (15)	0.0372 (16)	0.0302 (15)	0.0005 (13)	0.0074 (12)	0.0024 (12)
C17	0.0410 (16)	0.0426 (17)	0.0279 (15)	−0.0078 (14)	0.0029 (12)	0.0076 (13)
C18	0.0381 (15)	0.0286 (14)	0.0342 (15)	−0.0080 (13)	−0.0083 (12)	0.0053 (12)

### Geometric parameters (Å, °)

**Table:**

S1—C8	1.667 (2)	C6—C7	1.483 (3)
O1—C7	1.220 (3)	C9—C10	1.370 (3)
N1—C7	1.375 (3)	C9—C14	1.433 (3)
N1—C8	1.401 (3)	C10—C11	1.401 (4)
N1—H1	0.859 (16)	C10—H10A	0.9500
N2—C8	1.329 (3)	C11—C12	1.351 (4)
N2—C9	1.412 (3)	C11—H11A	0.9500
N2—H2	0.861 (16)	C12—C13	1.407 (4)
C1—C2	1.377 (4)	C12—H12A	0.9500
C1—C6	1.386 (3)	C13—C18	1.408 (4)
C1—H1A	0.9500	C13—C14	1.419 (3)
C2—C3	1.379 (4)	C14—C15	1.413 (3)
C2—H2A	0.9500	C15—C16	1.362 (3)
C3—C4	1.372 (4)	C15—H15A	0.9500
C3—H3A	0.9500	C16—C17	1.395 (4)
C4—C5	1.370 (4)	C16—H16A	0.9500
C4—H4A	0.9500	C17—C18	1.353 (4)
C5—C6	1.392 (3)	C17—H17A	0.9500
C5—H5A	0.9500	C18—H18A	0.9500
			
C7—N1—C8	128.1 (2)	C10—C9—N2	123.9 (2)
C7—N1—H1	119.1 (18)	C10—C9—C14	120.5 (2)
C8—N1—H1	112.8 (18)	N2—C9—C14	115.6 (2)
C8—N2—C9	132.4 (2)	C9—C10—C11	120.0 (3)
C8—N2—H2	112.6 (18)	C9—C10—H10A	120.0
C9—N2—H2	115.0 (18)	C11—C10—H10A	120.0
C2—C1—C6	120.3 (2)	C12—C11—C10	121.1 (3)
C2—C1—H1A	119.9	C12—C11—H11A	119.5
C6—C1—H1A	119.9	C10—C11—H11A	119.5
C1—C2—C3	120.1 (3)	C11—C12—C13	120.8 (3)
C1—C2—H2A	120.0	C11—C12—H12A	119.6
C3—C2—H2A	120.0	C13—C12—H12A	119.6
C4—C3—C2	120.0 (3)	C12—C13—C18	121.4 (2)
C4—C3—H3A	120.0	C12—C13—C14	119.5 (2)
C2—C3—H3A	120.0	C18—C13—C14	119.1 (2)
C5—C4—C3	120.4 (3)	C15—C14—C13	117.5 (2)
C5—C4—H4A	119.8	C15—C14—C9	124.4 (2)
C3—C4—H4A	119.8	C13—C14—C9	118.1 (2)
C4—C5—C6	120.3 (3)	C16—C15—C14	121.4 (2)
C4—C5—H5A	119.9	C16—C15—H15A	119.3
C6—C5—H5A	119.9	C14—C15—H15A	119.3
C1—C6—C5	119.0 (2)	C15—C16—C17	120.7 (3)
C1—C6—C7	124.2 (2)	C15—C16—H16A	119.7
C5—C6—C7	116.8 (2)	C17—C16—H16A	119.7
O1—C7—N1	121.8 (2)	C18—C17—C16	119.6 (3)
O1—C7—C6	120.7 (2)	C18—C17—H17A	120.2
N1—C7—C6	117.5 (2)	C16—C17—H17A	120.2
N2—C8—N1	114.9 (2)	C17—C18—C13	121.7 (3)
N2—C8—S1	128.4 (2)	C17—C18—H18A	119.2
N1—C8—S1	116.76 (19)	C13—C18—H18A	119.2
			
C6—C1—C2—C3	0.4 (4)	C14—C9—C10—C11	−0.6 (4)
C1—C2—C3—C4	0.9 (5)	C9—C10—C11—C12	2.2 (4)
C2—C3—C4—C5	−1.1 (5)	C10—C11—C12—C13	−1.7 (4)
C3—C4—C5—C6	0.1 (4)	C11—C12—C13—C18	−179.9 (2)
C2—C1—C6—C5	−1.5 (4)	C11—C12—C13—C14	−0.5 (4)
C2—C1—C6—C7	180.0 (2)	C12—C13—C14—C15	−178.2 (2)
C4—C5—C6—C1	1.2 (4)	C18—C13—C14—C15	1.2 (3)
C4—C5—C6—C7	179.9 (2)	C12—C13—C14—C9	2.0 (3)
C8—N1—C7—O1	−0.4 (4)	C18—C13—C14—C9	−178.6 (2)
C8—N1—C7—C6	180.0 (2)	C10—C9—C14—C15	178.8 (2)
C1—C6—C7—O1	165.4 (3)	N2—C9—C14—C15	−3.5 (4)
C5—C6—C7—O1	−13.2 (4)	C10—C9—C14—C13	−1.5 (3)
C1—C6—C7—N1	−15.0 (4)	N2—C9—C14—C13	176.2 (2)
C5—C6—C7—N1	166.4 (2)	C13—C14—C15—C16	−0.5 (4)
C9—N2—C8—N1	179.7 (2)	C9—C14—C15—C16	179.2 (2)
C9—N2—C8—S1	−0.6 (4)	C14—C15—C16—C17	−0.4 (4)
C7—N1—C8—N2	3.1 (4)	C15—C16—C17—C18	0.7 (4)
C7—N1—C8—S1	−176.7 (2)	C16—C17—C18—C13	0.0 (4)
C8—N2—C9—C10	−10.9 (4)	C12—C13—C18—C17	178.4 (3)
C8—N2—C9—C14	171.5 (3)	C14—C13—C18—C17	−0.9 (4)
N2—C9—C10—C11	−178.1 (2)		

### Hydrogen-bond geometry (Å, °)

**Table:**

*D*—H···*A*	*D*—H	H···*A*	*D*···*A*	*D*—H···*A*
N2—H2···O1	0.86 (2)	1.85 (2)	2.600 (3)	144 (2)
N1—H1···S1^i^	0.86 (2)	2.80 (2)	3.591 (2)	153 (2)
C15—H15A···O1	0.95	2.51	3.411 (3)	159.

Symmetry codes: (i) −*x*+1, −*y*+1, −*z*+1.
